# Statutory health insurance-covered pre-exposure prophylaxis in Germany: changing trends in nationwide tenofovir disoproxil/emtricitabine prescriptions during the COVID-19 pandemic

**DOI:** 10.3389/fphar.2023.1241310

**Published:** 2023-11-03

**Authors:** Henrieke Prins, Achim Dörre, Daniel Schmidt

**Affiliations:** ^1^ Department of Infectious Disease Epidemiology, Robert Koch Institute, Berlin, Germany; ^2^ ECDC Fellowship Programme, Field Epidemiology Path (EPIET), European Centre for Disease Prevention and Control (ECDC), Stockholm, Sweden

**Keywords:** pre-exposure prophylaxis (PrEP), pharmacy prescriptions, COVID-19, lockdown, Germany

## Abstract

**Background:** In 2019, Germany introduced a law to reimburse high-incidence populations for pre-exposure prophylaxis (PrEP), prescribed as tenofovir-disoproxil/emtricitabine (TDF/FTC), via statutory health insurance (SHI). We studied changes in TDF/FTC-prescriptions after the implementation of this law and during the COVID-19 pandemic.

**Methods:** We performed an interrupted time series analysis with monthly prescriptions per defined time period as the outcome. We considered the introduction of SHI-covered PrEP (09/2019) as an interruption, and four COVID-19 waves and two national lockdowns (2020–2021) as explanatory variables. We extrapolated prescriptions had the lockdowns not occurred, and compared this to the actual prescriptions. We performed sub-analyses based on stratification by five federal states with the highest proportion of PrEP users. We assessed the models’ goodness-of-fit based on the adjusted *R*-squared using RStudio.

**Results:** The best fitting model included SHI-covered PrEP and the first COVID-19 lockdown (04/2020). The decrease in prescriptions during the first lockdown was significant nationally, and in the five federal states for single-month prescriptions. The first lockdown resulted in reductions of 57.7% (95% prediction interval (PI): 23.0%–92.4%) for single-month prescriptions, while 17.4% (95% PI: 0.28%–34.5%) nationally, and 13.9% (95% PI: -3.67%–31.5%) for 3-month prescriptions.

**Conclusion:** Introduction of SHI-covered PrEP resulted in a doubling of TDF/FTC-prescriptions nationwide in the first month alone. A drop in prescriptions was most apparent after the first lockdown, and particularly affected PrEP initiations, possibly due to reduced healthcare access and behavioural changes. Ongoing monitoring of TDF/FTC-prescriptions is needed to safeguard access to preventative care such as PrEP and particularly PrEP initiation during public health crises like COVID-19.

## Introduction

Human immunodeficiency virus (HIV) pre-exposure prophylaxis (PrEP) based on daily oral antiretroviral medication is a safe and effective preventive measure that, when taken as prescribed, strongly reduces the risk of HIV acquisition among persons at risk (Grant et al., 2010; McCormack et al., 2016). PrEP is a vital part of the strategy to reach the UNAIDS goals for the AIDS epidemic by 2030 (Joint United Nations Programme on HI V/AIDS, 2015). Since 2016, PrEP has become increasingly available through healthcare systems in countries in Western and Central Europe (The Global PrEP Tracker, 2022; Monitoring Implementation of the Dublin Declaration, 2023).

Formally, a clinical assessment is needed prior to initiating PrEP. During treatment with PrEP, it is recommended that individuals pay regular healthcare visits for PrEP counselling, and that clinicians regularly follow up on renal function, and test for HIV and other sexually transmitted infections (STIs) (Spinner et al., 2019).

In Germany, PrEP became officially available in 2016 when the European Medicines Agency approved the use of tenofovir disoproxil and emtricitabine (TDF/FTC) for PrEP (Truvada to enhance existing HIV prevention strategies, 2016). However, the high costs of approximately 800 Euros per month in the absence of an affordable generic equivalent in Germany, or any legal option to import it from abroad, likely kept individuals who needed PrEP from using it.

In July 2017, PrEP became more affordable when the Truvada^®^ (TDF/FTC) patent expired. Still, costs of approximately 240 Euros per month in the case of continuous use (Emtricitabine/tenofovir disoproxil, 2022) remained high. From October 2017, costs for PrEP dropped to approximately 50 Euros per month after a pharmacist exploited a rarely used clause of the German regulatory framework (PrEP, 2017). Although this helped to make PrEP more affordable for some people (Ahaus et al., 2022), adequate counselling and testing was not yet widely accessible at that time.

In September 2019, Germany introduced a national law to reimburse populations at increased risk of acquiring HIV for PrEP, including counselling and testing, via statutory health insurance (SHI) (Gesetz für schnellere Termine und, 2019). In Germany, where health insurance is compulsory, 87% of citizens are covered by SHI (Busse et al., 2014; Thomas, 2017; Bundesministerium für Gesundheit, 2022). Since the implementation of the law, all German SHI companies cover the costs of PrEP when prescribed by qualified PrEP prescribers. As of June 2020, approximately 16,000 to 22,000 individuals were taking PrEP in Germany (Marcus et al., 2022).

The global COVID-19 pandemic emerged in early 2020. Subsequent national and regional public health measures such as lockdown policies, and COVID-19-related reductions of outpatient hours affected the availability of health services, and personal concerns about possible SARS-CoV-2 exposure affected social and healthcare-seeking behaviours (New South Wales Ministry of Health (NSW), 2020; Cowling et al., 2020; Pagaoa et al., 2021; Sentis et al., 2021; Ullrich et al., 2021). This in turn impacted PrEP demand and uptake (Schmidt et al., 2021a; Schmidt et al., 2022a; Schmidt et al., 2022b). However, the extent to which this pandemic period affected PrEP use in Germany remains largely unknown.

The objective of this study is to examine the changes in TDF/FTC prescription trends in Germany (country-wide and by federal states) after the implementation of SHI-covered PrEP in September 2019, and during the COVID-19 pandemic until December 2021. Understanding trends in PrEP prescriptions over time can help guide public health efforts to facilitate access to PrEP and ensure that PrEP coverage is successfully expanded. Moreover, it can help prevent potential negative effects of future pandemic responses on successful preventive health measures such as PrEP.

## Methods

### Data source

In Germany, medical prescriptions are electronically recorded, since SHI reimburses pharmacies via specialized pharmacy billing centres. From September 2019 onwards, data on TDF/FTC prescribed on SHI included prescriptions for PrEP. To include the period prior to SHI-covered PrEP, monthly prescription data on TDF/FTC in Germany were obtained from January 2018 to December 2021. The data were provided by Insight Health™, which claims >99% coverage of the SHI prescription market (≈73 million people). The data on the sale of TDF/FTC prescriptions from pharmacies originate from a database that is not person-specific and cannot be linked to other health insurance data or treatment indication. No accessible national data source for the SHI system currently exists, which would allow the validation of prescriptions according to treatment indication. The analysed anonymous prescription data included, for each month between January 2018 to December 2021: substance name, pack size, number of prescriptions, and location of prescription reimbursement. The data used here have a specific legal status in Germany and can be purchased under the regulations of the German Social Code Book V (§300 SGB V) with a delay of approximately 3 months.

### Assumptions prior to analysis

The only drug currently approved for PrEP in Germany is TDF/FTC, which individuals take as a fixed dose combination tablet. TDF/FTC is also used for HIV therapy and postexposure prophylaxis (PEP), in which cases it is combined with a third compound. Since our dataset of pharmacy billing data is anonymous and aggregated, there is no information on how often TDF/FTC was combined with a third compound. Therefore, TDF/FTC in this data source may have been prescribed for PrEP, HIV therapy, or PEP. However, we made attributions based on the ratio of prescription shares, previously stable trends in TDF/FTC as part of HIV therapy, and the knowledge that the standard treatment duration for PEP is 28–30 days. Moreover, HIV therapy is usually prescribed as a 3-month package, while PrEP is usually initiated with a single-month package, followed by 3-month TDF/FTC packages. We therefore analysed single-month and 3-month data separately.

In terms of the ratio of TDF/FTC prescription shares, the overall number of PEP treatments relative to PrEP as well as HIV therapy is low. In the recent years before SHI-covered PrEP, the number of TDF/FTC prescriptions slightly decreased. Recent analyses of unpublished, routine health insurance data used for surveillance purposes also suggest that the number of TDF/FTC prescriptions as part of HIV therapy remained relatively constant after the introduction of SHI-covered PrEP in September 2019. Hence, we assume that any potential abrupt changes in TDF/FTC prescriptions after September 2019 were mainly due to changes in PrEP use.

Since HIV treatment involves vital, lifelong therapy that must be taken daily, we assumed that HIV therapy was continued throughout the COVID-19 pandemic. Therefore, with respect to potential changes during the COVID-19 waves and lockdowns, we assumed that in the case of 3-month data, these changes primarily represented alterations in continued PrEP use, while changes in the single-month data primarily represent alterations in PrEP initiation, and to a lesser extent, in PEP.

### Analysis

We conducted an interrupted time series approach involving regression methods in which we modelled the implementation of SHI-covered PrEP as an interruption. The four COVID-19 waves and first two corresponding national lockdowns in Germany between January 2020 and December 2021 were included as potential explanatory variables in the regression models.

We defined the time periods as follows (Schilling et al., 2022): First wave: March to May 2020; second wave: October 2020 to February 2021; third wave: March to June 2021; fourth wave: August to December 2021. The first wave was accompanied by a strict national lockdown during April 2020. The second national lockdown started in November 2020 and ended in February 2021. Subsequent COVID-19 waves were not accompanied by strict national lockdowns.

Generally, the regression model was defined as follows accounting for either i) waves or ii) lockdowns:

i) TDF_FTC_*t*_ = Intercept + SHI_coverage_*t*_ + slope_before_SHI × *t* + slope_after_SHI × *t* + COVID_wave_*t*_
ii) TDF_FTC_*t*_ = Intercept + SHI_coverage_*t*_ + slope_before_SHI × *t* + slope_after_SHI × *t* + COVID_lockdown_*t*_

Where TDF_FTC_*t*_: TDF and FTC prescriptions in month *t*; SHI_coverage_*t*_: shift in prescriptions if PrEP is covered by SHI in month *t*; slope_before_SHI: monthly change of TDF/FTC prescriptions before PrEP was covered by SHI; slope_after_SHI: monthly change of TDF/FTC prescriptions after the law change that SHI covers PrEP; COVID_wave_*t*_: shift in prescriptions if month *t* is within the first COVID-19 pandemic wave; COVID_lockdown_*t*_: shift in prescriptions if month *t* is within the first COVID-19 COVID-19 lockdown.

The estimated shift in prescriptions directly after SHI coverage of PrEP is based on the difference between, respectively, the upper and lower ≥95% confidence interval bounds of the estimated mean number of prescriptions before and after the SHI coverage of PrEP in September 2019. The monthly changes before and after SHI coverage of PrEP were included as slopes in the regression models. The outcome was the number of monthly TDF/FTC prescriptions in Germany per defined period of time.

The hypothetical monthly prescription data had the four COVID-19 waves or two lockdowns not taken place were determined as linear extrapolations and subsequently compared to the actual observed number of prescriptions during the same time period. To assess potential changes during the four waves and two lockdowns, linear regression models were considered in which the interruptions were represented as binary explanatory variables, defined as 1 during the corresponding timespan of the pandemic wave or lockdown, and 0 if outside this timespan.

We performed sub-analyses based on single-month (28 tablets, 30 tablets, or 35 tablets) and 3-month data (84 tablets or 90 tablets), and stratified by the 16 German federal states (Baden-Wurttemberg, Bavaria, Berlin, Brandenburg, Bremen, Hamburg, Hesse, Mecklenburg-Western-Pomerania, Lower Saxony, North Rhine-Westphalia, Rhineland-Palatinate, Saarland, Saxony, Saxony-Anhalt, Schleswig-Holstein and Thuringia). We also compared separate trend analyses for the different regions and treatment durations (single-month versus 3-month prescriptions). For this purpose, we considered the five federal states with the largest metropoles in Germany where the proportion of PrEP users was highest in 2020: Berlin/Brandenburg, Bavaria, Hamburg, Hesse, North Rhine-Westphalia (Schmidt et al., 2022a). Berlin and Brandenburg were combined in this analysis, because people living with HIV in Brandenburg often tend to be treated at HIV care centres in Berlin (HIV/AIDS in Brandenburg, 2022).

We assessed the overall goodness-of-fit of the considered models based on the adjusted R-squared. Likewise, the adjusted R-squared was used to compare and choose among potential model variations. We conducted all analyses using R and RStudio.

### Ethical statement

Ethical approval and informed consent were not required, because we used routinely collected, anonymized secondary data from pharmacies, which cannot be traced back to individual patients. The German Social Code Book V (§300 SGB V) ensures safe use of this data.

## Results

**FIGURE 1:**
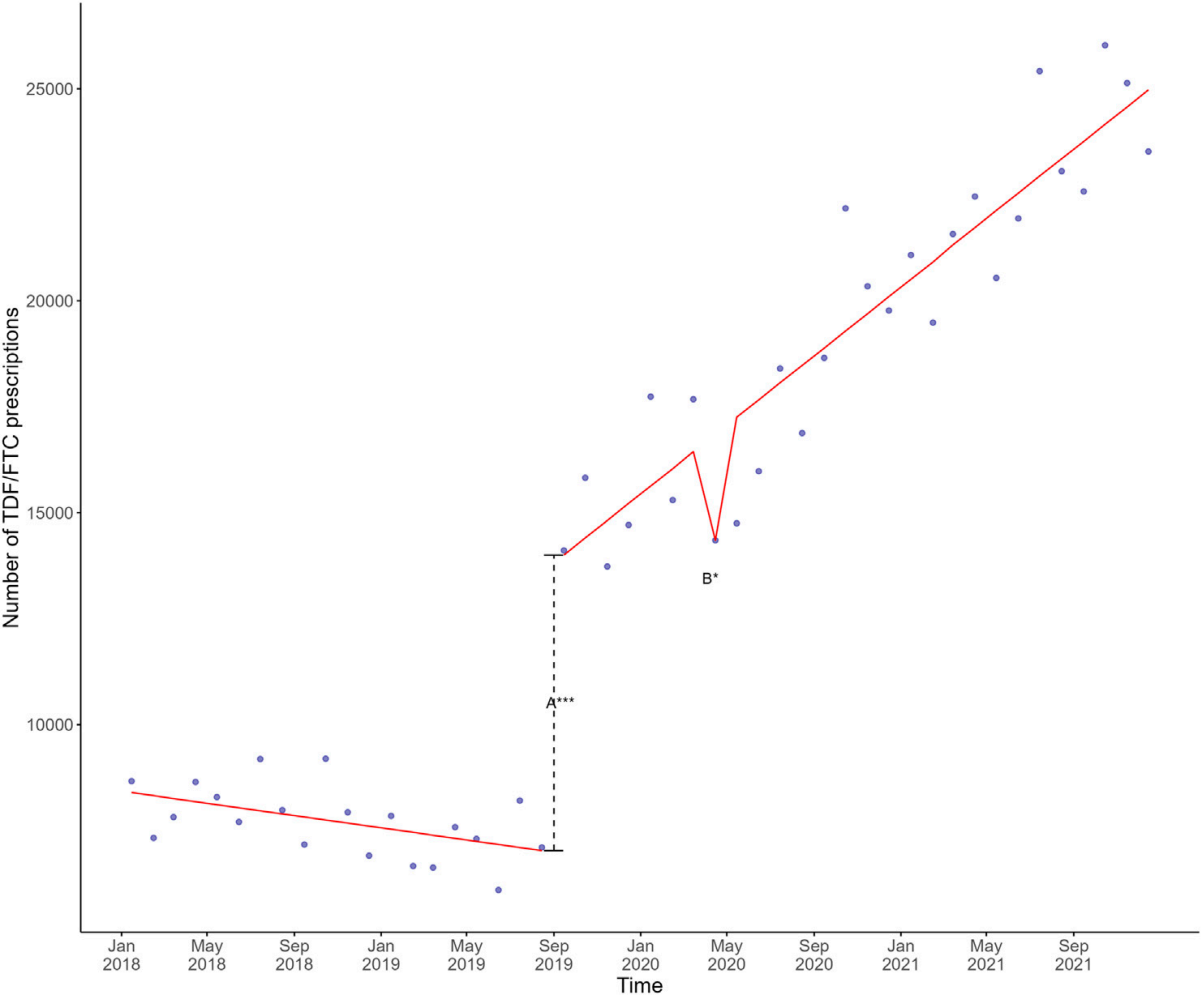
Mean estimates based on a linear regression model in red involving the shift directly after SHI coverage of PrEP (A) and the monthly change after the first lockdown (B) plotted against the actual observed monthly TDF/FTC prescriptions in blue in Germany, 2018–2021. The dashed line represents the shift directly after SHI coverage of PrEP. Significance codes: ***: 0–0.001; **: 0.001–0.01; *: 0.01–0.05. PrEP, pre-exposure prophylaxis; SHI, statutory health insurance; TDF, tenofovir disoproxil; FTC, emtricitabine.

From January 2018 to December 2021, there were a total of 697,555 TDF/FTC prescriptions in Germany (depicted as blue dots in Figure 1). From January to August 2018, the total number of monthly TDF/FTC prescriptions for HIV treatment and PEP declined by 8% from 8,670 to 7,986. With the introduction of SHI-covered PrEP in September 2019, prescriptions of TDF/FTC doubled from 7,105 to 14,113 (+99%) within 1 month. From October 2019 until March 2020, TDF/FTC prescriptions increased from 15,824 to 17,677 (+12%). In April 2020, TDF/FTC prescriptions declined to 14,352 (−19% compared to March 2020), followed by a slight increase in May and June 2020, with 18,402 prescriptions in July 2020. Despite monthly fluctuations, TDF/FTC prescriptions further increased during 2020 and 2021 with peaks in October 2020 (22,182) and October 2021 (26,030). In December 2021, TDF/FTC prescriptions were at 23,523.

**FIGURE 2:**
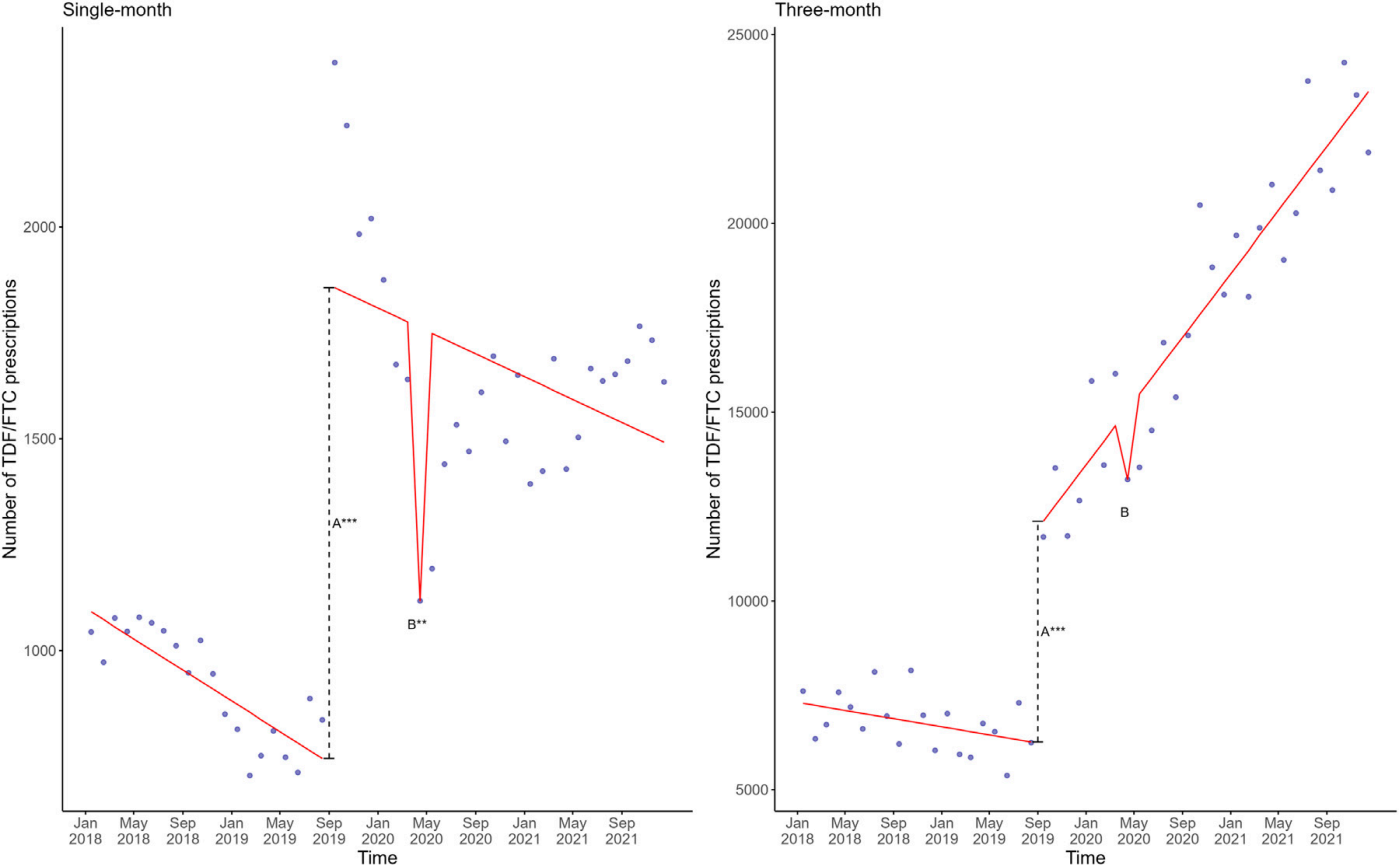
Mean estimates based on a linear regression model in red involving the shift directly after SHI coverage of PrEP (A) and the monthly change after the first lockdown (B) plotted against the actual observed single-month (left) and 3-month (right) TDF/FTC prescriptions in blue in Germany, 2018–2021. The dashed line represents the shift directly after SHI coverage of PrEP. Significance codes: ***: 0–0.001; **: 0.001–0.01; *: 0.01–0.05. PrEP, pre-exposure prophylaxis; SHI, statutory health insurance; TDF, tenofovir disoproxil; FTC, emtricitabine.

The different linear regression models involving SHI-covered PrEP, the four COVID-19 waves, and the two national lockdowns all exhibited acceptable goodness-of-fit with the actual prescription data. The best fitting linear regression model, depicted as a red line in Figure 1, included the variables SHI coverage of PrEP and the first COVID-19 lockdown, which were both statistically significant (Supplementary Appendix Table SA1). Plotting this model against the actual number of single-month and 3-month prescriptions showed a similar trend for 3-month prescriptions, but not for single-month prescriptions (Figure 2).

The percentage difference between the modelled number of TDF/FTC prescriptions if the first COVID-19 lockdown had not occurred, versus the actual number of TDF/FTC prescriptions was similar for the overall prescriptions (single- and 3-month prescriptions), and 3-month prescriptions: 2,496 for the overall TDF/FTC prescriptions (17.4% reduction, 95% prediction interval 0.28%–34.5%), and 1,843 for the 3-month prescriptions (13.9% reduction, 95% prediction interval −3.67%–31.5%). However, for the single-month prescriptions, this was 645 (57.7% reduction, 95% prediction interval 23.0%–92.4%).

**FIGURE 3:**
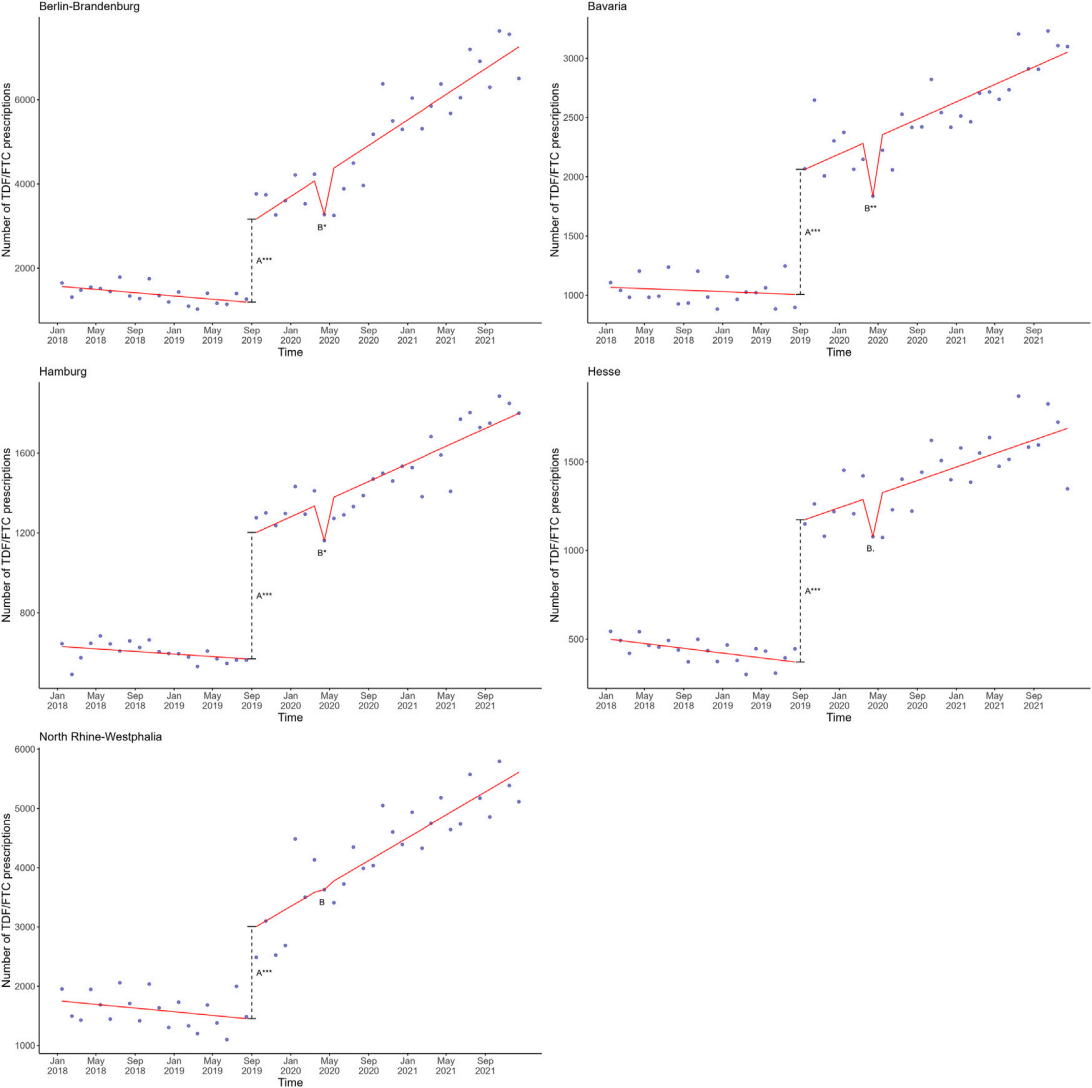
Mean estimates based on a linear regression model in red involving the shift directly after SHI coverage of PrEP (A) and the monthly change after the first lockdown (B) plotted against the actual observed monthly TDF/FTC prescriptions in blue in the five federal states with the largest metropoles in Germany, 2018–2021. The dashed line represents the shift directly after SHI coverage of PrEP. Significance codes: ***: 0–0.001; **: 0.001–0.01; *: 0.01–0.05. PrEP, pre-exposure prophylaxis; SHI, statutory health insurance; TDF, tenofovir disoproxil; FTC, emtricitabine.

The models assessing SHI-coverage of PrEP and the first COVID-19 wave and lockdown for the five federal states with the largest metropolitan cities, all exhibited acceptable goodness-of-fit with the actual data from these five federal states. The best fitting linear regression model for the five federal states included the first COVID-19 lockdown (Supplementary Appendix Table SA2), similar to the overall data at the national level (Supplementary Appendix Table SA1). When plotting this linear regression model against the actual number of observed TDF/FTC prescriptions for the five federal states, overall trends appeared largely similar except for North Rhine-Westphalia (Figure 3). The association with the first lockdown in the federal states Berlin-Brandenburg, Bavaria and Hamburg was statistically significant, and weakly statistically significant in Hesse (*p* = 0.0595) (Supplementary Appendix Table SA2). We did not observe a pronounced association with the first lockdown in North Rhine-Westphalia (Figure 3 and Supplementary Appendix Table SA2).

Following the analysis of the TDF/FTC prescription data for the five federal states with the largest metropoles, we assessed the number of single-month and 3-month prescriptions for each of these federal states using the same linear regression models. Generally, these models exhibited acceptable goodness-of-fit with the actual single-month and 3-month data from these five federal states. The linear regression model with the best fit included the variables SHI coverage of PrEP and the first wave, closely followed by the model including SHI-covered PrEP and the first lockdown (Supplementary Appendix Table SA3).

**FIGURE 4:**
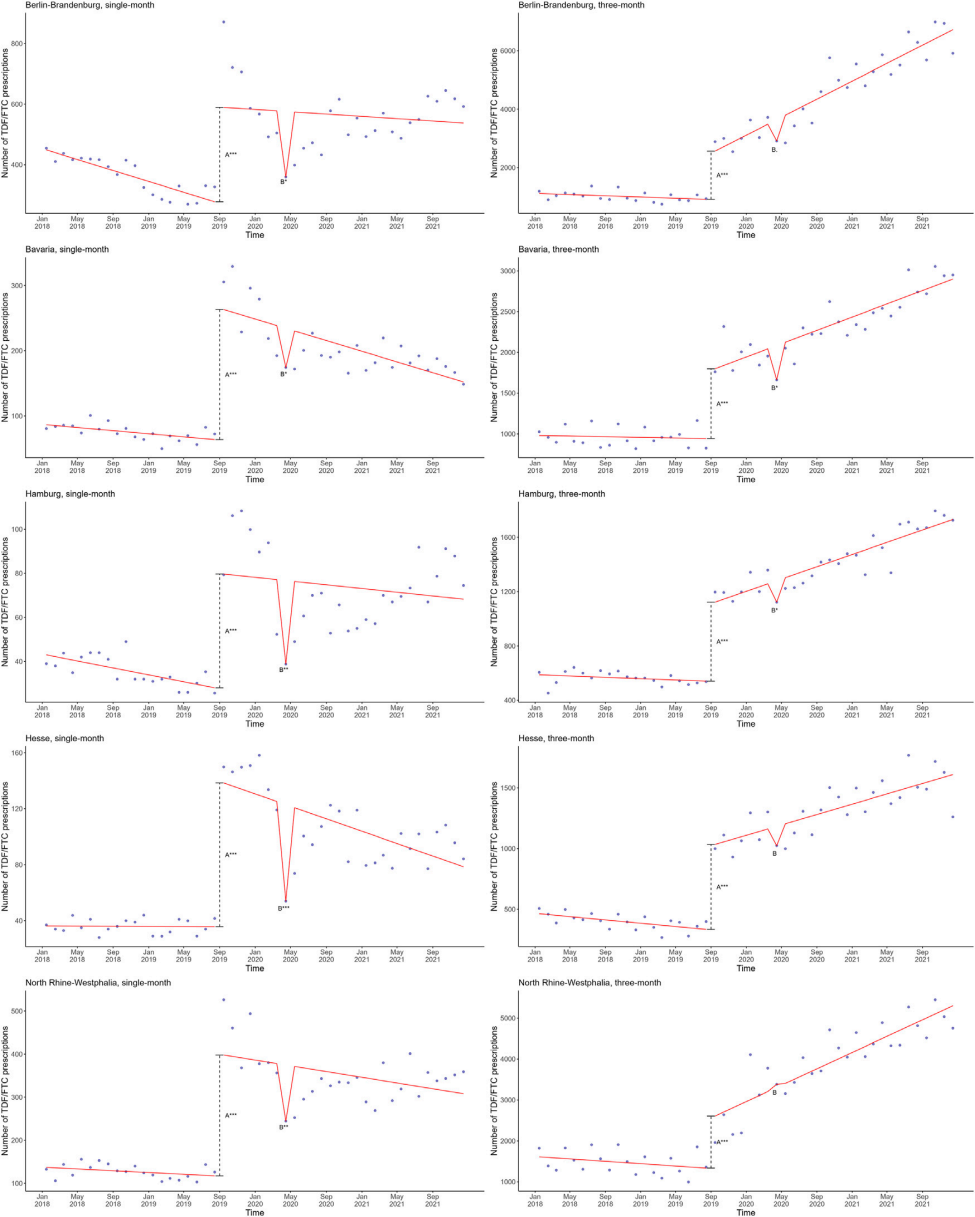
Mean estimates based on a linear regression model in red involving the shift directly after SHI coverage of PrEP (A) and the monthly change after the first lockdown (B) plotted against the actual observed single-month (left) and 3-month (right) TDF/FTC prescriptions in blue in the five federal states with the largest metropoles in Germany, 2018–2021. The dashed line represents the shift directly after SHI coverage of PrEP. Significance codes: ***: 0–0.001; **: 0.001–0.01; *: 0.01–0.05. PrEP, pre-exposure prophylaxis; SHI, statutory health insurance; TDF, tenofovir disoproxil; FTC, emtricitabine.

Finally, we plotted the linear regression models including SHI coverage of PrEP and the first COVID-19 lockdown against actual single-month and 3-month TDF/FTC prescriptions for the five federal states (Figure 4). The trends in single-month and 3-month prescriptions for these five regions appeared largely similar to the trends in single-month and 3-month prescriptions at the national level (Figure 2).

When comparing the five federal states to one another, the trends in single-month prescriptions appeared similar (Figure 4), and corresponds to a statistically significant association with the first lockdown in all five federal states (Supplementary Appendix Table SA3). Likewise, the trends in 3-month prescriptions appeared comparable to one another, with the exception of North-Rhine Westphalia (Figure 4), and corresponded to a statistically significant association with the first lockdown in most (Berlin-Brandenburg, Bavaria, Hamburg), but not all federal states (Hesse, North Rhine-Westphalia) (Supplementary Appendix Table SA3).

However, in each of the five federal states, the single-month and 3-month trends clearly differed. Although in both cases we observed a slightly declining trend from January 2018 to August 2019 (before the SHI coverage of PrEP), the subsequent increase was markedly more pronounced for the single-month data compared to the 3-month data (Figure 4). Moreover, the decrease in prescriptions coinciding with the first COVID-19 lockdown in April 2020 was more pronounced and statistically significant in each federal state for both the first COVID-19 wave and first lockdown for the single-month, but not the 3-month data (Supplementary Appendix Table SA3). Furthermore, following the SHI coverage of PrEP, there was an overall increase in 3-month prescription data versus a slightly decreasing trend in single-month data, similar to the trends observed at the national level (Figures 2, 4).

## Discussion

This study addresses changes in TDF/FTC prescriptions in Germany from January 2018 to December 2021. During this 4-year period, an important national law establishing the right for individuals at substantial risk of acquiring HIV to receive insurance-covered PrEP was implemented in September 2019. In the months that followed, the COVID-19 pandemic struck, resulting in several successive COVID-19 waves and lockdowns in Germany.

Our data show a remarkable jump in TDF/FTC prescriptions in the same month that SHI-covered PrEP was introduced in September 2019. Taken as a proxy for PrEP initiation, this clearly shows that being given the chance to start fully insurance-reimbursed PrEP motivated a large group of eligible persons to initiate this preventive treatment via their health insurance within 1 month of implementation of the law.

During the COVID-19 pandemic, the largest downward change of TDF/FTC prescriptions was observed during the first wave and lockdown in April 2020, similar to observations in France (Billioti de Gage et al., 2022). This is most likely explained by a combination of factors including changes in sexual risk behaviour during that time (PlanetRomeo, 2020; Sentis et al., 2021; Schmidt et al., 2022b), contact reduction due to the closing of nightclubs, bars and sex-on-premises venues (Graaf, 2020), but also decreased availability of resources and use of healthcare, which has also been reported in other European settings (Sentis et al., 2021). This effect was not visible in our data during subsequent waves or lockdowns. In Germany, surveys among HIV-specialty care centres conducted as part of a national PrEP evaluation project showed a decrease in PrEP initiations and an increase in PrEP interruptions during the first lockdown, and a subsequent increase in PrEP use during the summer of 2020 (Schmidt et al., 2021a; Schmidt et al., 2022a), similar to findings from a prospective observational study (Uhrmacher et al., 2022). Furthermore, in line with the absence of an effect during subsequent COVID-19 waves, a number of studies in similar settings indicate that the COVID-19 pandemic only temporarily affected sexual behaviour among certain vulnerable groups (Newman and Guta, 2020; de Sousa et al., 2021; van Bilsen et al., 2021), and that changes in PrEP use during the pandemic largely corresponded to both sexual behaviour associated with HIV risk and the extent of public restrictions (de la et al., 2022). Importantly, whether a decrease in TDF/FTC prescriptions was a consequence of reduced access to care, or a reduction in risk behaviour, or which of these is the most likely explanation cannot be inferred from this study’s prescription data alone.

This study shows that the most notable change during the first lockdown was on the single-month packages (rather than on the 3-month packages), both at the national level and in the five federal states with the largest German metropoles with the highest proportion of PrEP users. This implies that the first lockdown affected persons initiating PrEP more severely than persons who were continuing PrEP or HIV therapy. This is indeed likely, assuming that implementation of insurance-reimbursed PrEP caused a sharp increase in persons who initiated PrEP, and given that during the first wave, nationwide pandemic control measures and restrictions were in place which affected the federal states in much the same way.

From September 2019 until December 2021, overall trends in single-month prescription data decreased while 3-month prescriptions increased, most likely as a result of fewer new PrEP initiations and more PrEP and HIV therapy continuations. Possibly, the overall downward trend in PrEP initiations is also associated with the commonly given advice to reduce social contacts as part of restrictive measures in place during the four lockdowns that occurred between September 2019 and December 2021. Notably, North Rhine-Westphalia was the only federal state where we did not observe a significant association in the overall trend with the first lockdown, and in the federal states Hesse and North Rhine-Westphalia, the first lockdown significantly affected single-month TDF/FTC prescriptions, but not 3-month prescriptions, indicating that although PrEP initiations decreased during that time, the continuation of PrEP and HIV therapy was in fact maintained in these federal states. This drop in PrEP demand in particular for PrEP initiations is similar to what was reported by HIV specialty care centres in North Rhine-Westphalia, Hesse, and other federal states in surveys about PrEP availability, demand and uptake during the COVID-19 pandemic (based on analyses of previously published data (Schmidt et al., 2021b; Schmidt et al. 2020; ESchmidt et al., 2021)). However, further research is needed to understand the factors that could explain the findings. Importantly, the fact that an effect of our modelled interruptions or random shocks on TDF/FTC prescriptions was not observed does not necessarily mean that there was no effect, nor does it imply evidence of the absence of an effect in a real-life setting.

To effectively prevent HIV, PrEP requires compliant use for which continued access is important. Previous research found that PrEP use and adherence decreased after the implementation of pandemic-related control measures. Other research found that HIV testing rates decreased during the COVID-19 pandemic (Delaney et al. 2021; DiNenno et al., 2022) which might be partly explained by a reduction in PrEP prescriptions (Huang et al., 2022). To avoid missing HIV elimination targets, it is essential to provide continued PrEP care, even in times of public health crises. This is particularly true as it becomes evident that the COVID-19 pandemic amplified socioeconomic inequalities in health (Wachtler et al., 2020). To safeguard continued preventive health services, sexual health services and low-threshold facilities should remain operational as much as possible, and work to maintain a reputation as being safe and accessible to the public (Ullrich et al., 2021). Other strategies to continue providing steady care to this population include the expansion of telemedicine such as telehealth PrEP consultations with clinic pill pickups or home delivery (Nagendra et al., 2020; Hill et al., 2021), HIV self-testing or self-sample collection (Huang et al., 2022), or provision of PrEP through community-based organizations (Monitoring Implementation of the Dublin Declaration, 2023).

Robust surveillance and monitoring systems both within and outside Germany, which include data collection on the extent of informal online access to PrEP and health outcomes of existing monitoring are important, and indeed are part of recent recommendations by the European Centre for Disease Control (ECDC) as well as part of a current project that aims to establish a continuous PrEP surveillance in Germany (Monitoring Implementation of the Dublin Declaration, 2023; Schmidt, 2022).

### Bias and limitations

The monthly aggregated TDF/FTC prescription data for PrEP, PEP, and HIV treatment in Germany used in this study do not represent individual prescription data, and would equal the monthly number of prescriptions only in case of daily use. Based on the ratio of prescription shares, treatment duration, and stable trends in TDF/FTC prescriptions as part of HIV therapy, the monthly changes after September 2019 in this dataset most likely primarily reflect changes in PrEP use. However, no accessible national data source for the SHI system currently exists in Germany, and TDF/FTC prescriptions as part of HIV therapy or PEP cannot be directly distinguished. As such, we were unable to validate prescriptions according to treatment indication. Therefore, these data are an indirect measure and might under- or overestimate actual PrEP use. Within the dataset it is not possible to formally distinguish between new prescriptions and refills, nor can these data be linked to any demographic or behavioural data.

Furthermore, in October 2020 a reference price level regulation for TAF/FTC came into effect in Germany (Beschluss, 2023), which could potentially result in co-payment by HIV positive persons and therefore lead to an increase in TDF/FTC in HIV treatment. However, single tablet regimens are exempt from this reference price level regulation. As we solely consider TDF/FTC alone in the drug prescription data only HIV treatment switches to a non-single tablet regimen containing TDF/FTC would impact our data. Studies show that in recent years, single tablet regimens are mainly used in HIV treatment (Rüsenberg et al., 2021; Lottes et al., 2022; Zöllner and Pieloth, 2023). Therefore, the effect on the total amount of TDF/FTC prescriptions would be rather small, and regimen switches were likely spread out over multiple months resulting in more gradual changes in TDF/FTC prescriptions.

## Conclusion

In summary, PrEP use in Germany doubled after the establishment of the legal right to SHI-covered PrEP in September 2019. Since then, continuous PrEP use in Germany has steadily increased, although to what extent PrEP use would have increased in the absence of the COVID-19 pandemic cannot be known. Based on these data, it appears that only the first COVID-19 wave and corresponding lockdown negatively affected PrEP prescriptions, possibly as a result of reduced access to care and a reduction in risk behaviour. PrEP initiations were most negatively affected during the first lockdown, and to a lesser extent, PrEP continuation and HIV therapy.

The public health relevance of this finding is that in times of infectious disease outbreaks, maintenance of access to preventative care such as PrEP, and particularly PrEP initiation, merits attention. To this end, expanding PrEP coverage, such as through telemedicine, targeted communication, and low-threshold, outreach-based programs is vital. In conclusion, ongoing monitoring of trends in prescription, coupled with the assessment of contextual factors, is needed to investigate possible long-term effects, and to understand how PrEP use changes over time. This can guide future efforts to expand PrEP coverage, and help avoid lapses in access to PrEP in responses to future pandemics.

## Data Availability

Aggregated data supporting the conclusion of this article will be made available by the authors, upon reasonable request.
