# Supplementary material for: Statutory health insurance-covered pre-exposure prophylaxis in Germany: changing trends in nationwide tenofovir disoproxil/emtricitabine prescriptions during the COVID-19 pandemic
Source: Front Pharmacol. 2023 Nov 3;14:1241310. doi: 10.3389/fphar.2023.1241310 (PMC10654745; doi:10.3389/fphar.2023.1241310)
Supplement: Supplementary file 1 [file DataSheet1.pdf]

APPENDIX

Table 1. Results of two linear regression models with the best fit to the actual data, based on the estimate and *p*-value per variable, and adjusted *R*-squared

**Variable in linear regression model**	**Estimate**		**Adjusted *R*-squared**	
	First wave	*First lockdown*	*First wave*	*First lockdown*
Intercept	8473.0***	8473.0***	0.9661	0.9668
Shift directly after SHI coverage of PrEP	7101.3***	6972.3***		
Monthly change before SHI PrEP	-72.1	-72.1		
Monthly change after SHI PrEP	401.0***	406.5***		
Monthly change after first wave/first lockdown	-1345.8	-2496.5*		

Significance codes: ***: 0–0.001; **: 0.001–0.01; *: 0.01–0.05. PrEP: pre-exposure prophylaxis; SHI: statutory health insurance.

Table 2. Results of two linear regression models per federal with the best fit to the actual data, based on the estimate and *p*-value per variable, and adjusted *R*-squared

Federal state	Variable in linear regression model	Estimate		Adjusted *R*-squared	
		First wave	*First lockdown*	*First wave*	*First lockdown*
**BE/BB**	*Intercept*	1584 .4***	1584.4***	0.9599	0.9579
	Shift directly after SHI coverage of PrEP	2069.1***	1970.5***		
	Monthly change before SHI PrEP	- 19.5	-19.5		
	Monthly change after SHI PrEP	147.3***	151.5***		
	Change during first wave/first lockdown	-710.1*	-952.6*		
**BY**	*Intercept*	1070.1***	1070.1***	0.9546	0.9561
	Shift directly after SHI coverage of PrEP	1082.7***	1055.5***		
	Monthly change before SHI PrEP	-3.1	-3.1		
	Monthly change after SHI PrEP	35.5***	36.7***		
	Change during first wave/first lockdown	-268.9*	-482.0**		
**HH**	*Intercept*	633.8***	633.8***	0.9704	0.9726
	Shift directly after SHI coverage of PrEP	636.1***	632.9***		
	Monthly change before SHI PrEP	-3.2	-3.2		
	Monthly change after SHI PrEP	22.0***	22.1***		
	Change during first wave/first lockdown	-77.7	-195.5*		
**HE**	*Intercept*	505.9***	505.9***	0.9505	0.9514
	Shift directly after SHI coverage of PrEP	814.4***	802.2***		
	Monthly change before SHI PrEP	-6.7	-6.7		
	Monthly change after SHI PrEP	18.6***	19.1***		
	Change during first wave/first lockdown	-125.2	-229.0		
**NW**	*Intercept*	1763.5***	1763.5***	0.9361	0.9361
	Shift directly after SHI coverage of PrEP	1533.5***	1551.8***		
	Monthly change before SHI PrEP	-15.5	-15.5		
	Monthly change after SHI PrEP	97.3***	96.5***		
	Change during first wave/first lockdown	55.9	-51.1		

Significance codes: ***: 0–0.001; **: 0.001–0.01; *: 0.01–0.05. PrEP: pre-exposure prophylaxis; SHI: statutory health insurance. BE: Berlin, BB: Brandenburg, BY: Bavaria, HH: Hamburg, HE: Hesse, NW: North Rhine-Westphalia.

Table 3. Results of two linear regression models per federal for single-month and three-month TDF/FTC prescriptions with the best fit to the actual data, based on the estimate and *p*-value per variable, and adjusted *R*-squared

**Federal state**	**Variable in linear regression model**	**Estimate**				**Adjusted *R*-squared**			
		First wave		*First lockdown*		*First wave*		*First lockdown*	
		Single-month	Three-month	Single-month	Three-month	Single-month	Three-month	Single-month	Three-month
**BE/BB**	*Intercept*	458.2***	1126.3***	458.2***	1126.3***	0.676	0.9627	0.6355	0.9617
	Shift directly after SHI coverage of PrEP	336.8***	1720.0***	311.6***	1647.2***				
	Monthly change before SHI PrEP	-9.0**	-10.5	-9.0**	-10.5				
	Monthly change after SHI PrEP	-3.0	150.8***	-1.9	153.9***				
	Change during first wave/first lockdown	-172.7***	-533.2*	-216.3*	-729.8				
**BY**	*Intercept*	87.7***	982.2***	87.7***	982.2***	0.9153	0.9539	0.8889	0.9565
	Shift directly after SHI coverage of PrEP	210.0***	872.6***	199.3***	856.1***				
	Monthly change before SHI PrEP	-1.2	-2.0	-1.2	-2.0				
	Monthly change after SHI PrEP	-4.6***	40.1***	-4.1***	40.8***				
	Change during first wave/first lockdown	-62.3***	-206.4	-60.1*	-421.7*				
**HH**	*Intercept*	43.8***	590.0***	43.8***	590.0***	0.7328	0.972	0.6708	0.974
	Shift directly after SHI coverage of PrEP	56.9***	579.2***	51.6***	581.3***				
	Monthly change before SHI PrEP	-0.8	-2.5	-0.8	-2.5				
	Monthly change after SHI PrEP	-0.6*	22.6***	-0.4	22.6***				
	Change during first wave/first lockdown	-33.7***	-44.0	-37.9**	-157.6*				
**HE**	*Intercept*	36.3***	470.1***	36.3***	470.1***	0.8758	0.9475	0.8703	0.9483
	Shift directly after SHI coverage of PrEP	108.1***	706.6***	102.7***	699.8***				
	Monthly change before SHI PrEP	-0.02	-6.8	-0.02	-6.8				
	Monthly change after SHI PrEP	-2.4***	21.0***	-2.2***	21.3***				
	Change during first wave/first lockdown	-44.4***	-80.1	-68.9***	-159.4				
**NW**	*Intercept*	138.0***	1625.4***	138.0***	1625.4***	0.8773	0.9245	0.8632	0.9238
	Shift directly after SHI coverage of PrEP	295.0***	1238.4***	280.7***	1271.1***				
	Monthly change before SHI PrEP	-1.0	-14.5	-1.0	-14.5				
	Monthly change after SHI PrEP	-4.0***	101.2***	-3.3**	99.8***				
	Change during first wave/first lockdown	-100.3***	155.7	-130.3**	77.4				

Significance codes: ***: 0–0.001; **: 0.001–0.01; *: 0.01–0.05. PrEP: pre-exposure prophylaxis; SHI: statutory health insurance; TDF: tenofovir disoproxil; FTC: emtricitabine. BE: Berlin, BB: Brandenburg, BY: Bavaria, HH: Hamburg, HE: Hesse, NW: North Rhine-Westphalia
